# Rodent-borne diseases and their public health importance in Iran

**DOI:** 10.1371/journal.pntd.0006256

**Published:** 2018-04-19

**Authors:** Mohammad Hasan Rabiee, Ahmad Mahmoudi, Roohollah Siahsarvie, Boris Kryštufek, Ehsan Mostafavi

**Affiliations:** 1 Department of Epidemiology, Faculty of Veterinary Medicine, University of Tehran, Tehran, Iran; 2 Department of Epidemiology and Biostatistics, Research Centre for Emerging and Reemerging Infectious Diseases, Pasteur Institute of Iran, Tehran, Iran; 3 National Reference Laboratory for Plague, Tularemia and Q fever, Research Centre for Emerging and Reemerging Infectious Diseases, Pasteur Institute of Iran, Akanlu, Iran; 4 Department of Biology, Faculty of Sciences, Ferdowsi University of Mashhad, Mashhad, Iran; 5 Rodentology Research Department (RRD), Institute of Applied Animal (IAA), Ferdowsi University of Mashhad, Mashhad, Iran; 6 Slovenian Museum of Natural History, Ljubljana, Slovenia; Yale University, UNITED STATES

## Abstract

**Background:**

Rodents are reservoirs and hosts for several zoonotic diseases such as plague, leptospirosis, and leishmaniasis. Rapid development of industry and agriculture, as well as climate change throughout the globe, has led to change or increase in occurrence of rodent-borne diseases. Considering the distribution of rodents throughout Iran, the aim of this review is to assess the risk of rodent-borne diseases in Iran.

**Methodology/Principal finding:**

We searched Google Scholar, PubMed, Science Direct, Scientific Information Database (SID), and Magiran databases up to September 2016 to obtain articles reporting occurrence of rodent-borne diseases in Iran and extract information from them. Out of 70 known rodent-borne diseases, 34 were reported in Iran: 17 (50%) parasitic diseases, 13 (38%) bacterial diseases, and 4 (12%) viral diseases. Twenty-one out of 34 diseases were reported from both humans and rodents. Among the diseases reported in the rodents of Iran, plague, leishmaniasis, and hymenolepiasis were the most frequent. The most infected rodents were *Rattus norvegicus* (16 diseases), *Mus musculus* (14 diseases), *Rattus rattus* (13 diseases), *Meriones persicus* (7 diseases), *Apodemus* spp. (5 diseases), *Tatera indica* (4 diseases), *Meriones libycus* (3 diseases), *Rhombomys opimus* (3 diseases), *Cricetulus migratorius* (3 diseases), and *Nesokia indica* (2 diseases).

**Conclusions/Significance:**

The results of this review indicate the importance of rodent-borne diseases in Iran. Considering notable diversity of rodents and their extensive distribution throughout the country, it is crucial to pay more attention to their role in spreading infectious diseases for better control of the diseases.

## Author summary

This review showed that approximately half of the known rodent-borne diseases have been reported in Iran, half of which were reported both in humans and rodents. Most of the diseases were bacterial and parasitic. Plague, leishmaniasis, and hymenolepiasis were the most frequent diseases among rodent populations. Also, this review showed that among the rodent species, three commensal ones—*R*. *norvegicus*, *M*. *musculus*, and *R*. *rattus*—play an important role in the transmission of diseases to humans in Iran. Considering repeated reports of many of these diseases in humans and rodents, and the notable diversity and extensive distribution of rodents throughout Iran, it is crucial to pay adequate attention to rodents as a source of zoonotic infectious diseases in the country.

## Introduction

Rodents are the largest order of living mammals, comprising approximately 42% of global mammalian biodiversity [1, 2]. With almost 2,277 known species in 33 families, rodents have nearly a worldwide distribution, being absent only from Antarctica and some isolated islands [2]. They are characterized by a peculiar dentition consisting of a single pair of continuously growing incisors in each of the upper and lower jaws and a set of chewing teeth [2, 3]. Brandt (1855) on the basis of the zygomasseteric structure divided rodents into 3 suborders: Sciuromorpha, Hystricomorpha, and Myomorpha. This classification, even though widely accepted, has been also a matter of dispute [2, 4, 5]. Several years later, Wilson and Reeder adopted a 5-suborder system, i.e., Sciuromorpha, Castorimorpha, Anomaluromorpha, Hystricomorpha, and Myomorpha, among which the last suborder is the biggest in terms of species richness and population numbers [2]. This suborder contains more than half of rodents’ species and almost a quarter of the identified mammalian species [1]. Rodents are small- to medium-sized mammals, with short reproductive cycle and large litters, as well as morphological and biological adaptations to different lifestyles (e.g., terrestrial, subterranean, gliding, etc.) and environments (e.g., semiaquatic, aquatic, or dry biotopes). This high compatibility makes rodents one of the best suited mammals for living in various habitats [1, 3]. In spite of rodents’ beneficial activities such as soil aeration, mineral nutrient cycling, increase in water absorption, facilitation of biotic recovery, and control of insect populations, they can cause significant economic losses (primarily through feeding on stored food) and increase health risk by transmitting various infectious agents to humans [6]. Indeed, rodents are well-known reservoirs and hosts for a number of infectious diseases (e.g., plague, leptospirosis, leishmaniasis, salmonellosis, and viral hemorrhagic fevers) and play an important role in their transmission and spreading [7].

Rodent-borne diseases fall into one of two main categories: directly or indirectly transmitted diseases. In the former category, diseases are transmitted by being bitten or by inhaling the germ in feces of rodents, whereas in the latter category, humans are infected as the result of consuming food and water contaminated by rodent feces or urine. Likewise, rodents could act as amplifier hosts in the case of diseases transmitted by arthropod vectors from rodents to humans. Furthermore, rodents accidentally eaten by livestock could mediate disease transmission to humans if products of these livestock were not treated properly prior to consumption [8].

Iran is located between 25 to 40 degrees northern latitude and 44 to 63 degrees eastern longitude. Due to low latitude, aridity, and high fluctuation of daily and annual temperature, Iran has a variety of climate systems. Namely, the major mountain ridges (Alborz and Zagros) [9, 10] along with sever climatic influences of the Caspian Sea in the north and the Persian Gulf in the south have led to the formation of four major climatic regions in the Iranian Plateau: mild and humid (the southern beaches of the Caspian Sea), cold (western mountains), hot and dry (central part of the Iranian Plateau), and hot and humid (southern seashores: Persian Gulf and Sea of Oman) [11]. The diverse topography and different ecological conditions of Iran made it a corridor for the faunal exchanges between Asia and Europe [12, 13] and at the same time a speciation zone for a number of rodents (e.g., *Allactaga*, *Microtus*, *Mus*) [14–16].

Several studies have shown that rodents, particularly the suborder Myomorpha, have scattered widely in Iran [12, 17, 18]. So far, about 79 species of rodents have been identified in the country, with the following species being the most widespread: *Allactaga elater*, *Jaculus blanfordi*, *Microtus socialis*, *Gerbillus nanus*, *Meriones crassus*, *M*. *libycus*, *M. persicus*, *Rhombomys* *opimus*, *Tatera* *indica*, *Apodemus witherbyi*, *Mus* *musculus*, *Nesokia* *indica*, *Rattus* *norvegicus*, *R*. *rattus*, *Dryomys nitedula*, and *Hystrix indica* [18, 19]. House mice (*M*. *musculus*) and rats (*R*. *rattus*, *R*. *norvegicus*) occupy various habitats at greater density than the other species and pose considerable problems [20, 21]. Dipodidae and Gerbilinae dominate the arid and semiarid regions (e.g., *A*. *elater*, *Jaculus* spp., *T*. *indica*, *Gerbillus* spp., *Meriones* spp., and *R*. *opimus*). Arvicolinae is the dominant group in the mountains, grasslands, cultivated areas, and river valleys in western Iran (e.g., species of *Microtus*, *Arvicola*, and *Chionomys*). The genus *Apodemus* occupies different habitats, reaching highest diversity in northern parts of the country [6, 22–24]. On the other hand, occurrence of rodent-borne diseases have been documented in virtually all provinces of Iran [22]. Despite this, no attempt has been made to compare occurrence of these diseases together and assess the risk of each of these diseases. Therefore, the aim of this review is to assess the risk of rodent-borne diseases in Iran by reviewing the Iranian and international publications on occurrence of these diseases in rodents and humans throughout the country.

## Methods

This study is a review article in which the articles indexed in Google Scholar, PubMed, Science Direct, Scientific Information Database (SID), and Magiran databases were searched up to September 2016. First, we browsed the databases to obtain articles that indicate which infectious diseases are rodent-borne, using keywords like “rodent-borne diseases,” “rodent-borne pathogens,” “mouse-borne diseases,” and “rat-borne diseases.” Then, rodent-borne disease names were extracted from identified literature [7, 8, 25–34]. Afterwards, we browsed the databases to obtain articles reporting occurrence of rodent-borne diseases in rodents and humans in Iran. Keywords were “extracted rodent-borne disease names, Iran,” “extracted rodent-borne disease names, rodents, Iran,” “bacteria, rodent, Iran,” “parasite, rodent, Iran,” “virus, rodent, Iran,” “bacteria, mouse, Iran,” “parasite, mouse, Iran,” and “virus, mouse, Iran.” In addition, references of the selected articles were also reviewed to increase the scope of search and to cover all the related articles. Rodent-borne diseases in terms of infectious agents of diseases are divided into 3 groups of bacterial, viral, and parasitic diseases; concerning each disease, data related to infectious agents of disease and history of the disease report in human and rodents in Iran were extracted from the articles. Eventually, data on reported and unreported diseases in Iran were written in tables, separately.

## Results

Results of our review showed that among 70 worldwide known rodent-borne diseases, 34 were reported from Iran, out of which 17 (50%), 13 (38%), and 4 (12%) were parasitic, bacterial, and viral, respectively (Tables 1, 2, 3, and 4). Among these diseases, 21 were reported in both humans and rodents, including *Escherichia coli* enteritis, salmonellosis, plague, yersiniosis, leptospirosis, campylobacteriosis, tularemia, tick-borne relapsing fever, tuberculosis, Crimean–Congo hemorrhagic fever, cryptosporidiosis, toxoplasmosis, leishmaniasis, hepatic capillariasis, trichinellosis, gongylonemiasis, hymenolepiasis, taeniasis, alveolar echinococcosis, trichuriasis, and moniliformiasis. Bartonellosis, babesiosis, and plagiorchiasis have been reported only in rodents, while 10 diseases—listeriosis, Lyme disease, Q fever, hepatitis E, rabies, hemorrhagic fever with renal syndrome, toxocariasis, giardiasis, schistosomiasis, and fascioliasis—have only been documented in humans.

**Table 1:** Bacterial rodent-borne diseases reported in Iran.

Disease	Agent	Reports in Iran					Reference
		Human report		Rodent report			
		Number	Province	Number	Species	Province	
***E*. *coli* enteritis**	*E*. *coli*	>10	Golestan, Tehran, Fars, Khuzestan, Hamadan, Sistan- Baluchestan, Yazd, Ardebil	2	*Rattus* *rattus*, *R*. *norvegicus*	Tehran, Gilan	[35–39]
**Salmonellosis**	*Salmonella* spp.	>10	Goletan, Fars, Tehran, Mazandaran, Yazd, Ardebil, Khorasan, Khuzestan	4	*Mus* *Musculus*, *R*. *rattus*, *R*. *norvegicus*	Tehran, Gilan	[35–38, 40, 41]
**Plague**	*Yersinia pestis*	>10	Kurdistan, East Azerbaijan	>10	*Meriones* *persicus*, *M*. *libycus*, *Meriones vinogradovi*, *Meriones tristrami*	Kurdistan, Hamadan	[38, 42–46]
**Yersiniosis**	*Y*. *pseudotuberculosis*, *Y*. *enterocolitica*	2	Tehran, Golestan	1	*Rattus* *rattus*, *R*. *norvegicus*	Gilan	[36, 47, 48]
**Leptospirosis**	*Leptospira interrogans*	>10	Gilan Mazandaran, Golestan, Sistan-Baluchetsan Kerman, Tehran, Fars, Chaharmahal, Khuzestan, West Azerbaijan	4	*Nesokia* *indica*, *Mus* *musculus*, *Rattus* *rattus*, *R*. *norvegicus*, *Apodemus* spp., *Meriones* *libycus*, *Rhombomys* *opimus*	Khorasan, Khuzestan, Mazandaran	[49–53]
**Campylobacteriosis**	*Campylobacter* spp.	>10	Mazandaran, Golestan, Tehran, East Azerbaijan, Fars, Khuzestan, Lorestan, Kermanshah, Khorasan	1	*Sciurus anomalus*	Chaharmahal, Isfahan	[35, 38, 54]
**Tularemia**	*Francisella tularensis*	3	Kurdistan, Sistan-Baluchestan	2	*Microtus paradoxus*, *Tatera* *indica*	Golestan, Sistan- Baluchestan	[55–60]
**Tick-borne relapsing fever**	*Borrelia* spp.	>10	Ardebil, Hamadan, Zanjan, Kurdistan, Qazvin, Fars, Hormozgan	1	*Rattus* *norvegicus*	Hormozgan	[61–69]
**Tuberculosis**	*Mycobacterium tuberculosis* complex	>10	AP	2	*Mus* *musculus*	West Azerbaijan	[70–75]
**Bartonellosis**	*Bartonella* spp.	0	-	1	*Mus* *musculus*	Hamadan	[76]
**Listeriosis**	*Listeria* spp.	2	Tehran, Fars	0	*-*	-	[77, 78]
**Lyme disease**	*Borrelia burgdorferi*	5	Tehran, Mazandaran	0	-	-	[79–82]
**Q fever**	*Coxiella burnetii*	>10	Mazandaran, Khuzestan, Khorasan, Semnan, Kerman, Fars, Kurdistan, Tehran	0	-	-	[83–87]

**Abbreviation:** AP, All Provinces

**Table 2:** Viral rodent-borne diseases reported in Iran.

Disease	Agent	Report in Iran					Reference
		Human report		Rodent report			
		Number	Province	Number	Species	Province	
**Hepatitis E**	Hepatitis E virus	>10	Kermanshah, Hamadan, East Azerbaijan, Isfahan, Khuzestan, Chaharmahal	0	-	-	[88]
**Rabies**	Rabies virus	>10	AP	0	-	-	[89]
**Crimean–Congo hemorrhagic fever**	Nairovirus	>10	AP	2	*Allactaga williamsi*, *Mus* *musculus*, *Meriones* *crassus*	-	[38, 90, 91]
**HFRS**	Hantaan virus, Puumala virus, Dobrava virus, Seoul virus	1	Isfahan	0	-	-	[92]

**Abbreviations:** AP, All Provinces; HRFS, hemorrhagic fever with renal syndrome.

**Table 3:** Parasitic rodent-borne diseases reported in Iran.

Disease	Agent	Report in Iran					Reference
		Human report		Rodent report			
		Number	Province	Number	Species	Province	
**Cryptosporidiosis**	*Cryptosporidium* spp.	>10	AP	4	*Mus* *norvegicus*, *R*. *rattus*, *Mus* *musculus*,	Hormozgan, Tehran, Khuzestan	[69, 93–98]
**Toxoplasmosis**	*Toxoplasma gondii*	>10	AP	5	*Rattus* *rattus*, *R*. *norvegicus*	Gilan, Khuzestan, Tehran, Kohgiluyeh- Boyerahmad	[99–104]
**Leishmaniasis**	*Leishmania infantom*, *Leishmania major*, *Leishmania tropica*, *Leishmania donovani*	>10	AP	>10	*Meriones* *persicus*, *Cricetulus* *migratorius*, *M*. *libycus*, *Rhombomys* *opimus*, *Tatera* *indica*, *Nesokia* *indica*, *Gerbillus* sp., *M. hurrianae*, *Mesocricetus brandti*, *Rattus* *rattus*, *Mus* *musculus*, *R*. *norvegicus*	Ardebil, Isfahan, Semnan, Yazd, Fars, Golestan, Sistan-Baluchestan, Hormozgan	[105–122]
**Hepatic capillariasis**	*Capilaria hepatica*	1	Tehran	3	*Meriones* *persicus*, *Mus* *musculus*, *Rattus* *rattus*, *R*. *norvegicus*, *Cricetulus migratorius*	Ardebil, Kermanshah	[123–126]
**Trichinellosis**	*Trichinella* spp.	2	Tehran	1	*Meriones* *persicus*	Isfahan	[127, 128]
**Hymenolepiasis (Rodentolepiasis)**	*Rodentolepis nana*, *Rodentolepis diminuta*	>10	AP	>10	*Rattus* *rattus*, *R*. *norvegicus*, *Mus* *musculus*, *Rhombomys* *opimus*, *Tatera* *indica*, *Apodemus* spp., *Meriones persicus*, *Microtus* *socialis*, *Cricetulus* *migratorius*,	Mazandaran, East Azerbaijan, Golestan, Sistan-Baluchestan, Hamadan, Isfahan, Khuzestan, Ardebil, Tehran, Kermanshah, Khorasan	[116, 124–126, 129–150]
**Taeniasis**	*Taenia* spp.	>10	Ardebil, Tehran, Arak, Mazandaran, Hamadan, Kermanshah	5	*Rattus* *norvegicus*, *R*. *rattus*, *Mus* *musculus*, *Apodemus* spp.	Mazandaran, East Azerbaijan, Hamadan, Kermanshah, Ardebil	[125, 126, 139, 142, 143, 145, 151–157]
**Alveolar echinococcosis**	*Echinococcus* *multilocularis*	2	Ardebil, Khorasan	1	*Microtus transcaspicus*, *Ochotona rufescens*, *Mus* *musculus*, *Crocidura gmelini*, *Apodemus* spp.	Khorasan	[158–160]
**Moniliformiasis**	*Moniliformis moniliformis*	4	Sistan-Baluchestan, Isfahan, Khorasan, Khuzestan	5	*Mus* *musculus*, *Rattus* *rattus*, *R*. *norvegicus*, *Meriones* *persicus*	East Azerbaijan, Khuzestan, Ardebil, Kerman	[124, 126, 143, 147, 150, 161–164]
**Trichuriasis**	*Trichuris* spp.	3	Khuzestan, Ardebil	6	*Mus* *musculus*, *Rattus* *norvegicus*, *R*. *rattus Tatera* *indica*	Kermanshah, Kerman, Hamadan, Golestan, Ardebil, Mazandaran	[116, 125, 126, 129, 144, 145, 150, 165, 166]
**Gongylonemiasis**	*Gongylonema* spp.	1	NS	3	*Rattus* *norvegicus*, *R*. *rattus*	Khuzestan, East Azerbaijan	[167–170]
**Babesiosis**	*Babesia* spp.	0	-	3	*Meriones* *persicus*, *Rattus* *norvegicus*, *Mus* *musculus*	Ardebil, Hormozgan, East Azerbaijan	[69, 171–173]
**Plagiorchiasis**	*Plagiorchis muris*, *P*. *hilippinensis*, *P*. *javanensis*	0	-	2	*Rattus* *norvegicus*, *Apodemus* spp.	Hamadan	[145, 174]
**Toxocariasis**	*Toxaocara* spp.	>10	Gilan, Tehran, Hamadan, Khuzestan, Zanjan, Mazandaran, Fars, Kermanshah	0	-	-	[175–183]
**Schistosomiasis**	*Schistosoma* spp.	>10	Khuzestan	0	-	-	[184, 185]
**Giardiasis**	*Giardia lamblia (G*. *duodenalis)*	>10	AP	0	-	-	[186]
**Fasciolosis**	*Fasciola hepatica*, *F*. *gigantica*	>10	Gilan, Mazandaran, Kermanshah, Kohgiluyeh- Boyerahmad, Ardebil	0	-	-	[187]

**Abbreviations:** AP, All Provinces; NS, Not Stated

**Table 4:** Rodent- borne diseases not yet reported in Iran.

	Disease	Agent
**Bacterial Diseases**	**Rickettsialpox**	*Rickettsia akari*
	**Sylvatic typhus**	*Rickettsia prowazekii*
	**Murine typhus**	*Rickettsia typhi*
	**Boutonneuse or Mediterranean spotted fever**	*Rickettsia conorii*
	**Rocky Mountain spotted fever**	*Rickettsia rickettsi*
	**Rat-bite fever**	*Spirillum minus*, *Streptobacillus moniliformis*
	**Human granulocytic anaplasmosis**	*Anaplasma phagocytophilum*
	**Corynebacteriosis**	Non-diphtheritic corynebacterium
	**Pasteurellosis**	*Pasteurella* spp.
**Viral Diseases**	**Rift Valley fever**	Rift Valley fever virus
	**HPS**	Hantaviruses
	**Borna disease**	Borna virus
	**Lassa fever**	Lassa virus
	**Argentine hemorrhagic fever**	Junin virus
	**Bolivian hemorrhagic fever**	Machupo virus
	**Lujo hemorrhagic fever**	Lujo virus
	**Venezuelan hemorrhagic fever**	Guanarito virus
	**Brazilian hemorrhagic fever**	Sabia virus
	**Kyasanur Forest Disease**	Kyasanur Forest disease virus
	**Omsk hemorrhagic fever**	Omsk hemorrhagic fever virus
	**Tick-borne encephalitis**	Tick-borne encephalitis virus
	**Apoi virus disease**	*Apoi virus*
	**Powassan encephalitis**	*Powassan virus*
	**Colorado tick fever**	*Coltivirus*
	**Cowpox**	Cowpox virus
	**Venezuelan Equine Encephalitis**	*Venezuelan equine encephalitis virus*
	**Western Equine Encephalitis**	*Western equine encephalitis virus*
	**Lymphocytic choriomeningitis**	Lymphocytic choriomeningitis virus
**Parasitic Diseases**	**Chagas**	*Trypanosoma cruzi*
	**Toxascariasis**	*Toxascaris leonina*
	**Baylisascariasis**	*Baylisascaris procyonis*
	**Angiostrongylosis**	*Angiostrongylus cantonensis*, *Angiostrongylus costaricensis*
	**Echinostomiasis**	*Echinostoma*
	**Alariasis**	*Alaria* spp.
	**Neosporosis**	*Neospora caninum*
	**Brachylaimiasis**	Brachylaimidae

**Abbreviation:** HPS, hantavirus pulmonary syndrome.

For 8 out of 34 diseases, rodents are known to be the primary or definitive host, including in plague, leptospirosis, tick-borne relapsing fever, Lyme disease, hemorrhagic fever with renal syndrome, leishmaniasis, hymenolepiasis, and moniliformiasis; meanwhile, in other reported diseases, rodents act as the secondary host.

Of these 34 diseases, 11 of them—plague, tularemia, tick-borne relapsing fever, bartonellosis, Lyme disease, Q fever, Crimean–Congo hemorrhagic fever, leishmaniasis, babesiosis, schistosomiasis, and fasciolosis—not only are rodent-borne but also are vector-borne diseases. The other 23 diseases are only rodent-borne.

Except plague, which has been only reported from the western part of Iran (Kurdistan, Hamadan, East Azerbaijan), and Schistosomiasis, which has been only reported from the southwestern region of the country (Khuzestan), the rest (32 diseases) were reported from various regions of Iran.

Out of 31 diseases reported from humans in Iran, 20 were reported frequently (more than 10 reports), and 11 were scarcely reported (fewer than 10 reports). The first category includes plague, *E*. *coli* enteritis, salmonellosis, leptospirosis, campylobacteriosis, tick-borne relapsing fever, Q fever, tuberculosis, hepatitis E, rabies, Crimean–Congo hemorrhagic fever, cryptosporidiosis, toxoplasmosis, leishmaniasis, hymenolepiasis, taeniasis, toxocariasis, schistosomiasis, giardiasis, and fasciolosis. The second category consists of yersiniosis (2 reports), tularemia (3 reports), listeriosis (2 reports), Lyme disease (5 reports), hemorrhagic fever with renal syndrome (1 report), hepatic capillariasis (1 report), trichinellosis (2 reports), alveolar echinococcosis (2 reports), moniliformiasis (4 reports), trichuriasis (3 reports), and gongylonemiasis (1 report).

Out of 24 reported diseases among rodents of Iran, the 3 diseases plague, leishmaniasis, and hymenolepiasis were the most frequently reported. These diseases had more than 10 reports, while other diseases—*E*.*coli* enteritis, salmonellosis, yersiniosis, leptospirosis, campylobacteriosis, tularemia, tick-borne relapsing fever, tuberculosis, bartonellosis, Crimean–Congo hemorrhagic fever, cryptosporidiosis, toxoplasmosis, hepatic capillariasis, trichinellosis, taeniasis, alveolar echinococcosis, moniliformiasis, trichuriasis, gongylonemiasis, babesiosis, and plagiorchiasis—had fewer than 10 reports among rodents of Iran.

Overall, based on the reviewed databases, 10 species of rodents in Iran are categorized as high-index infectious regarding the number of pathogens and diseases reported on them: *R*. *norvegicus* (16 diseases), *M*. *musculus* (14 diseases), *R*. *rattus* (13 diseases), *M*. *persicus* (7 diseases), *Apodemus* spp. (5 diseases), *T*. *indica* (4 diseases), *M*. *libycus* (3 diseases), *R*. *opimus* (3 diseases), *C*. *migratorius* (3 diseases), and *N*. *indica* (2 diseases).

## Discussion

This review showed that almost half of the known rodent-borne diseases (34 out of 70) so far have been reported in Iran, and out of the 34 diseases, 21 diseases were reported in both rodents and humans. Three diseases (i.e., bartonellosis, babesiosis, and plagiorchiasis) were only reported from rodents and may be listed as hazardous for human communities, too. Ten diseases—rabies, hemorrhagic fever with renal syndrome, listeriosis, Lyme disease, Q fever, hepatitis E, toxocariasis, giardiasis, schistosomiasis, and fasciolosis—were reported only from humans. However, since both infectious agents and rodent hosts of these diseases are present in Iran [19, 22, 23], rodents may act as possible mediators. Therefore, all of the reported rodent-borne diseases in Iran are important. Nevertheless, plague and leishmaniasis are of greatest concern because of their repeated reports in rodent and human populations, complicated transmission and maintenance cycle, and their pathogenesis that causes sever diseases in humans.

In the case of plague, although in recent years occurrence of the disease has not been reported in human populations, the disease was reported repeatedly in the past in Iran, and so far, several big epidemics of human plague occurred in Iran. During 1772–1773, one of the largest epidemics of plague in the world occurred in Iran and the area under control of Iran at that time. This epidemic led to the deaths of around 2 million people. Moreover, outbreaks of Plague were identified in rodents reservoirs, including in *M*. *persicus*, *M*. *libycus*, *M*. *vinogradovi*, and *M*. *tristrami*, in active foci of the plague in west Iran (Kurdistan Province) every 2–3 years [188]. Therefore, this disease should be monitored continuously in rodent populations of Iran, especially in western and northwestern areas, so that possible occurrence and emergence in human populations can be prevented.

In the case of leishmaniasis, occurrence of the disease for the first time was reported in visceral form in 1949, and since then the disease has been reported increasingly in different provinces of Iran. Currently, both forms of the disease (Cutaneous Leishmaniasis [CL] and Visceral Leishmaniasis [VL]) are endemic to Iran, and approximately 20,000 cases due to CL and 100–300 cases due to VL annually are recorded in Iran, although the actual number of CL may be 4 or 5 times higher [122, 189]. In addition, rodents are the main reservoir for the wet (rural) CL in Iran, and this infection has been reported frequently and circulated among rodents of Iran, especially *R*. *opimus*, *M*. *libycus*, *M*. *hurrianae*, *and T*. *indica* [188]. Therefore, the rodent reservoir of the wet CL in each endemic province should be tackled carefully so that the prevalence of the diseases in humans can be decreased effectively.

Although some of the rodent-borne diseases have not been thus far reported from Iran (Table 4), the rodent hosts [19, 22, 23, 190, 191] or intermediate hosts (vectors) of some of them (e.g., *Rhipicephalus sanguineus*, vector of Boutonneuse or Mediterranean spotted fever) exist in Iran [192–194]. In addition to this, some of these diseases (e.g., lymphocytic choriomeningitis) are occurring in Iran’s neighboring countries [195, 196]. Also, nowadays human activity and climate change can affect spatial distributions; annual/seasonal cycles; incidence and severity of many infectious diseases, particularly zoonotic ones; and thus they can lead to occurrence of emerging infectious diseases throughout the globe [8, 197]. It is, therefore, possible that those diseases eventually occur in rodents of Iran and can be transmitted to humans.

Rodent-borne viral diseases include approximately one-third of all known rodent-borne diseases [8, 198], and it was shown that their transmission and prevalence vary in different regions depending on various virus–host systems; environmental regulators; and anthropogenic, genetic, behavioral, and physiologic factors [199]. In fact, some of these diseases are more frequently reported in certain regions of the world. For example, hantavirus pulmonary syndrome (HPS) in the Latin America region has been reported repeatedly both in humans and rodents, while hemorrhagic fever with renal syndrome is more prevalent in Eurasia. Also, lymphocytic choriomeningitis is endemic to Europe and has been reported in humans and rodents of this region, especially in *M*. *musculus* [28, 200–204]. In Iran, most of the reported rodent-borne diseases have been parasitic or bacterial. Indeed, only 4 out of 34 diseases (hepatitis E, Rabies, Crimean–Congo hemorrhagic fever, hemorrhagic fever with renal syndrome) mentioned in this review have the viral agents. It seems that our current knowledge suffers from lack of relevant research of viral rodent-borne diseases in the country. Moreover, the tough effect of the global climate changes on the algorithm of viral zoonotic disease occurrence should be considered in future studies.

The present review also showed that 11 diseases in Iran were vector-borne, including plague, tularemia, tick-borne relapsing fever, bartonellosis, Lyme disease, Q fever, Crimean–Congo hemorrhagic fever, leishmaniasis, babesiosis, schistosomiasis, and fasciolosis. In other words, almost one-third of the reported rodent-borne diseases in Iran are transmitted by vectors. Most of the vectors are ticks, fleas, mosquitoes, and sand flies [205–208]. Snails are mediators for schistosomiasis and fasciolosis [188, 209]. It is therefore important to pay attention to ectoparasites of rodents, along with studying their own species.

*M*. *musculus*, *R*. *rattus*, and *R*. *norvegicus* hold apparently the first rank regarding the rodent-borne diseases in Iran. Indeed, more than half of the reported diseases in Iran are connected to these commensal species. However, one should keep in mind that insufficient surveys have been performed on noncommensal rodent species in Iran [6]. *M*. *persicus*, *Apodemus* spp., *T*. *indica*, *M*. *libycus*, *R*. *opimus*, *C*. *migratorius*, and *N*. *indica* held the second rank regarding reported diseases. Therefore, performing comprehensive studies on the prevalence of the diseases in all rodent species of the different regions of Iran is unavoidable. Indeed, it is necessary to study other rodent species as much as the commensals *M*. *musculus*, *R*. *rattus*, and *R*. *norvegicus*.

Studying the evolution of host–parasite interactions at spatiotemporal scales is a necessary complement to the study and control of infectious diseases. For this purpose, it is crucial to accurately identify the species of both parasites and their hosts. Indeed, cryptic species may vary in their habitats and host preferences [210], and misidentification of the species may lead to wrong interpretation of host–parasite interactions and their coevolution, with serious implications for human health [211]. Unfortunately, some of the previous studies on rodent-borne diseases in Iran suffer from species misidentification. For example, *Apodemus sylvaticus*, *Apodemus flavicollis*, and *Mesocricetus auratus* were repeatedly mentioned in the research performed on the rodent-borne studies of Iran [51, 145], while recent molecular studies revealed that these 3 species do not exist in Iran [13, 212, 213]. Moreover, not all the entities of undetected cryptic species complexes are harmful or cause trouble for human. Taxonomic identification is, therefore, economically important so that the resources will not be spent on nontarget species [210]. This purpose implicates the engagement of biosystematic integrative approaches such as molecular standard tools and morphological analyses. For instance, DNA barcoding has proven to be a good cut-off for many vectors’ delimitation of leishmaniasis [214]. Moreover, new advances in phylogenomic-scale sequence data (e.g., whole genome sequencing [WGS], short-read sequence [SRS], etc.) are remarkably increasing in the case of clinical or taxonomical perspectives that produce an extraordinary resolution for difficult problems [215, 216]. Recent papers indicate how much taxonomy and public health benefit from type-sequence analyses, and it seems that research can go much farther with full genomes of reference taxonomy isolates [217]. For instance, applying WGS approaches allows taxonomists to understand what makes each species unique, with important consequences for unbiased species delimitation. From a medical perspective, clinicians will be allowed to anticipate clinical symptoms of the cases and to screen for genes that are responsible for antimicrobial resistance (AMR) in human pathogens [217]. Therefore, new technologies will evidently revolutionize many disciplines (e.g., microbiology and taxonomy) in the near future. On the other hand, it has been shown that phenotypic variation is associated with parasite infection [218]. It implies that morphological studies and the assessment of the evolutionary consequences of phenotypic trait variation on host populations should be included in the integrative studies of host–parasite interactions.

Vast and rapid developments of industry and agriculture in the past century have caused an increase in food products and provided suitable shelters in urban regions for populations of urban mice in many developed and developing countries [6]. Moreover, as the ancient climate changes have left strong imprints on modern ecosystems, human-dominated recent climate changes will also affect the composition and distribution of biota in the future. Taking into account this possibility, any shift of the host species’ range in response to climate fluctuations may change the distribution of the parasites and the agents, which may tend to prominent alterations in trends of rodent-borne disease occurrence [8]. Thus, multidisciplinary collaboration among a vast range of experts (e.g., epidemiologist, rodentologist, entomologist, ecologist, and microbiologist) is unavoidable in rodent-borne disease research for better understanding the current and future hazardous places for these diseases.

In a nutshell, this review showed the importance of rodent-borne diseases in Iran, some of which have the potential to cause huge epidemics in human populations, such as plague. Therefore, it is necessary to seriously consider the role of rodents in spreading infectious diseases in Iran for their better control. Also, it is necessary to conduct more detailed and multidisciplinary studies on these diseases to better understand the occurrence of these diseases in Iran. Finally, the impact of climate change on the prevalence and distribution of the diseases, especially vector-borne diseases, should be studied so that the occurrence of emerging or re-emerging infectious diseases such as plague can be predicted and hopefully prevented.

**Key learning points**

- Almost half of the known rodent-borne diseases have been reported in Iran so far.
- Most of the reported rodent-borne diseases in Iran are parasitic and bacterial.
- Most of the rodent-borne diseases in Iran are reported both in rodents and humans.
- Plague, leishmaniasis, and hymenolepiasis are the most reported and important diseases in rodents of Iran.
- Three commensal species—*R*. *norvegicus*, *M*. *musculus*, and *R*. *rattus*—play an important role in the transmission of diseases to humans among the rodents in Iran.

**Top 5 papers**

1. Meerburg BG, Singleton GR, Kijlstra A. Rodent-borne diseases and their risks for public health. Crit Rev Microbiol. 2009;35(3):221–70. PubMed PMID: 19548807.
2. Kim B, Vincent H, Heikki H, Darouny P, Jean-Pierre H, Philippe B. Rodent-Borne Zoonotic Viruses in Southeast Asia. Kasetsart J. 2009;43:94–105.
3. Han BA, Schmidt JP, Bowden SE, Drake JM. Rodent Reservoirs of Future Zoonotic Diseases. Proc Natl Acad Sci U S A. 2015;112(22):7039–44. PubMed PMID: 26038558.
4. Herbreteau V, Bordes F, Jittapalapong S, Supputamongkol Y, Morand S. Rodent-borne Diseases in Thailand: Targeting Rodent Carriers and Risky Habitats. Infect Ecol Epidemiol. 2012;2. PubMed PMID: 22957129.
5. Gubler DJ, Reiter P, Ebi KL, Yap W, Nasci R, Patz JA. Climate variability and change in the United States: potential impacts on vector- and rodent-borne diseases. Environ Health Perspect. 2001;109(Suppl 2):223–33. PubMed PMID: 11359689.
